# Pain Acceptance Creates an Emotional Context That Protects against the Misuse of Prescription Opioids: A Study in a Sample of Patients with Chronic Noncancer Pain

**DOI:** 10.3390/ijerph18063054

**Published:** 2021-03-16

**Authors:** Rosa Esteve, Estefanía Marcos, Ángela Reyes-Pérez, Alicia E. López-Martínez, Carmen Ramírez-Maestre

**Affiliations:** Facultad de Psicología y Logopedia, Andalucía Tech, Instituto de Investigación Biomédica de Málaga, Universidad de Málaga, 29071 Málaga, Spain; zarazaga@uma.es (R.E.); psicologiaestefi@gmail.com (E.M.); angela_rp_@hotmail.com (Á.R.-P.); aelm@uma.es (A.E.L.-M.)

**Keywords:** opioid prescriptions, pain acceptance, pain catastrophizing, depression, chronic pain, misuse

## Abstract

There is solid evidence of an association between several psychological flexibility processes, particularly pain acceptance, and adaptation to chronic pain. However, there are relatively few studies on the relationship between pain acceptance and opioid misuse in chronic pain patients. Thus, the aim of the present study was to test a hypothetical model in which pain acceptance would regulate pain sensations and pain-related thoughts and emotions, which would be related to opioid misuse. The sample comprised 140 chronic pain patients attending two hospitals. All patients were receiving pharmacological treatment, including opioid analgesics. Structural equation modelling analyses showed a significant association between higher pain acceptance and lower pain intensity and catastrophizing, and lower levels of anxiety and depression. Only higher anxiety and depression were significantly associated with increased opioid misuse. The results suggest that levels of anxiety, depression, and pain acceptance must be assessed before opioids are prescribed. Pain acceptance implies a relationship with internal events that protects against anxiety and depression and thus against opioid misuse. Acceptance and Commitment Therapy appears to be particularly appropriate for these patients.

## 1. Introduction

Chronic pain is a public health problem. In Europe, an estimated 27% of the general adult population experiences this condition [1]. It has a strong negative impact; is one of the main causes of disability; and entails economic costs higher than those of heart disease, diabetes, and cancer together [2].

Opioid therapy is now used for a broad range of chronic pain conditions [3]. The gradual increase in the use of opioids has become a global phenomenon and is generating social concern. In a 2016 report, the International Narcotics Control Board (INCB) provided data on opioid misuse and its harmful effects [4]. It has been found that the use of long-term opioid treatment increases the risk of death due to unintentional overdose and cardiorespiratory problems [5]. Of note, almost half of the patients entering treatment for opioid use disorder reported that their first exposure to opioids was through a physician’s prescription for pain management [6]. We define the misuse of opioids as their use in a manner other than how they are indicated or prescribed [7]. Studies should be conducted on the factors underlying opioid misuse, including psychological ones, because opioid misuse can cause health problems and lead to deadly opioid overdose [8]. In summary, chronic pain and prescription opioid misuse constitute two relevant societal challenges.

From a biopsychosocial perspective, many factors can contribute to understanding the experience of chronic pain, including biophysical, psychological, social, and genetic factors, as well as comorbidities [9]. Since the advent of the biopsychosocial model of pain, much research has addressed the role of the psychological factors involved in adjustment to chronic pain [10]. The Cognitive Behavioral (CB) model of pain is a broad framework applied in treating chronic pain [11] and encompasses more specific models, such as the Flexibility Model [12]. Recently, the relevance of studying transdiagnostic psychological factors has been emphasized, including psychological flexibility, which could maintain and exacerbate chronic pain and opioid misuse [13].

Psychological flexibility has been defined as “the ability to contact the present moment more fully as a conscious human being, and to change or persist in behavior when doing so serves valued ends” [14] (p. 7). Acceptance and its opposite, experiential avoidance, are core processes of psychological flexibility [15,16]. Experiential avoidance refers to an affect-related regulatory process that involves avoiding upsetting emotions, thoughts, memories, and other private experiences, such that individuals attempt to alter the form and frequency of internal events [17]. In contrast, acceptance implies that individuals actively and consciously welcome these private events and do not attempt to change their frequency or form [14].

As a functional approach, the psychological flexibility model pulls together behaviors that are diverse in their surface features but equivalent in their psychological function. Thus, from this perspective, alcohol and drug abuse are conceived as forms of experiential avoidance since they can contribute to the control or elimination of unwanted private experiences [18]. Several studies have investigated the relationship between experiential avoidance and substance abuse. A specific instrument has even been developed to assess experiential avoidance in persons who misuse substances (i.e., the Acceptance and Action Questionnaire-Substance Abuse [AAQ-SA]) [19]. The Acceptance and Action Questionnaire (AAQ) [20] has been shown to have inadequate internal consistency when used to assess such individuals, and when they have been assessed with the AAQ, it has been shown that experiential avoidance does not mediate Acceptance and Commitment Therapy (ACT) outcomes in addiction [21,22]. The AAQ-SA includes specific items addressing thoughts, feelings, and urges related to substance abuse, has better internal consistency and, people reporting any substance abuse in the last 30 days obtained lower score on the AAQ-SA [19]. Contradictory results have been obtained on the association between experiential avoidance and substance use [23,24,25], which may be due to the aforementioned limitations of the assessment instruments. Evidence of the relationship between experiential avoidance and substance abuse has been obtained from meta-analyses of the efficacy of ACT [26,27]. In the treatment of substance abuse, significant small to medium effect sizes were obtained with ACT compared to those of active treatment (e.g., Cognitive Behavioral Therapy, pharmacotherapy, 12-step, or standard treatment).

There is considerable evidence supporting an association between several psychological flexibility processes—particularly pain acceptance—and adjustment to chronic pain [12]. Associations have been found between higher pain acceptance and lower levels of anxiety, depression, pain catastrophizing, pain intensity, and disability [28,29]. Moreover, pain acceptance has been associated with lower medication intake [30,31,32,33,34]. This finding is relevant given that there is a growing body of evidence to suggest that prescribed opioid medication misuse is alarmingly frequent in chronic pain patients [35,36,37,38].

To our knowledge, relatively few studies have explored the relationship between pain acceptance and opioid misuse in chronic pain patients. A study on a sample of the general population who were experiencing pain and had used painkillers in the month previous to the study found that persons who were less accepting of pain were at more risk of developing opioid dependence [39]. Another study assessed a sample of patients receiving residential addiction treatment for comorbid pain. After controlling for demographic and other risk factors, an association was found between higher pain acceptance and a lower likelihood of developing opiate use disorders [40]. A recent study on a sample of patients recruited from two outpatient pain clinics found that psychological flexibility mediated the association between pain severity and opioid misuse and between pain interference and opioid misuse [41]. Due to the paucity of studies and the contradictory results presented in the literature, further research is needed in order to shed light on the relationship between psychological flexibility and opioid misuse in patients with noncancer chronic pain.

Thus, the aim of the present study was to test a hypothetical model in a sample of patients receiving opioid therapy for noncancer chronic pain. Because pain acceptance implies the willingness of patients to come into direct contact with unpleasant experiences such as pain sensations or pain-related thoughts and emotions [42], we postulated that there would be an association between higher pain acceptance and lower pain intensity, lower pain catastrophizing, and lower levels of anxiety and depression [28,29] and an association between higher pain intensity, higher pain catastrophizing, higher anxiety, and depression and increased opioid misuse (i.e., pain acceptance would regulate pain sensations and pain-related thoughts and emotions, which would be associated with opioid misuse).

## 2. Materials and Methods

### 2.1. Participants

All participants were fully informed of the aim of the study and were assured of their personal anonymity and the confidentiality of the survey. Subsequently, their informed consent was obtained to voluntarily participate in the study.

A total of 147 patients were invited to take part in the study. Of these, five refused participation, and two did not meet the inclusion criteria. The recruitment process was conducted from October 2019 to February 2020, which finished at that time due to the COVID-19 lockdown measures. Individuals were considered eligible for inclusion if they met the following criteria: At the moment of participation in the study, they were experiencing pain and had been experiencing pain for at least the last 6 months; they were between 18 and 65 years old; they were not being treated for a malignancy, terminal illness, or psychiatric disorder; they were able to understand the Spanish language; and they were able to understand the instructions and questionnaires. The final sample comprised 140 chronic pain patients attending two hospitals. All participants were receiving pharmacological treatment, including opioid analgesics. In Structural Equation Modelling analyses, sample sizes of at least 10 cases per parameter are considered to be sufficient [43]. Therefore, the final sample size was satisfactory.

The project was conducted in accordance with the Declaration of Helsinki and received ethical clearance by the Institutional Ethics Review Board (ERC UMA-66-2019-H) and the Regional Hospital Ethics Committee.

### 2.2. Procedure

The patients were informed of the study aims, confidentiality was assured, and informed consent was obtained. Each participant then took part in a semi-structured interview with a psychologist to obtain demographic, social, and medical history data. Subsequently, they completed the different questionnaires. Data collection was conducted by two psychologists who had previously been trained in the application of the protocol to ensure the standardization of the assessment process.

### 2.3. Variables and Instruments

#### 2.3.1. Demographic and Clinical Variables

Each participant had a semi-structured interview with a psychologist to collect demographic, social, and medical history information.

#### 2.3.2. Pain Intensity

The participants were asked to rate their lowest, average, and worst pain during the previous week, as well as their current pain intensity level, on a numerical rating scale (NRS) ranging from 0 (“No pain”) to 10 (“Worst pain possible”). These ratings were then averaged into a single composite pain intensity score. Numerical rating scales are commonly used in pain research and are known to provide valid and reliable measures of pain intensity across different populations [44].

#### 2.3.3. Pain Acceptance

We applied the Spanish version of the questionnaire (CPAQ-SV) [45,46]. This instrument comprises 20 items. It is similar to the original questionnaire and provides a total score and two subscale scores for pain willingness and activity engagement. The CPAQ-SV shows good internal consistency (α = 0.83). Two studies on the CPAQ-SV [45,47] have supported the validity of the 20-item version. The CPAQ-SV also demonstrates good criterion validity. In this study, the total score showed excellent reliability (α = 0.91).

#### 2.3.4. Pain Catastrophizing

Catastrophizing is a cognitive process characterized by an expectation of negative outcomes and a lack of confidence and control [48]. It is considered to be a maladaptive coping strategy that intensifies the experience of pain [49].

The 2-item Coping Strategies Questionnaire (CSQ) [50] was used to assess pain catastrophizing. Respondents indicate the frequency with which they experienced two catastrophizing thoughts and feelings when in pain on a 7-point scale ranging from 0 (“Never”) to 6 (“Always”). This scale has been shown to provide a valid and reliable measure of catastrophizing when used with chronic pain patients [50]. In the current sample, the standardized alpha coefficient indicated good levels of reliability (α = 0.89).

#### 2.3.5. Anxiety and Depression Symptoms

The Hospital Anxiety and Depression Scale (HADS) [51,52] is a practical screening tool for identifying and quantifying anxiety and depression in nonpsychiatric patients attending medical outpatient clinics. It comprises 14 items and two subscales: anxiety and depression. Each subscale consists of seven items in which respondents indicate on a 4-point scale the frequency with which they experienced anxiety and depression symptoms. The Spanish version of the scale shows appropriate reliability and validity [52,53,54]. The internal consistency of both scales is high (α = 0.86 for anxiety and α = 0.86 for depression). In the current sample, the standardized alpha coefficient indicated good levels of reliability (α = 0.85 and 0.84 for the anxiety and depression scales, respectively).

#### 2.3.6. Current Misuse of Prescribed Opioids

We applied the Spanish translation of the Current Opioid Misuse Measure (COMM) [55,56]. This is a brief patient self-assessment instrument for monitoring chronic pain patients receiving opioid therapy. The COMM comprises 17 items rated from 0 = “never” to 4 = “very often”. It was developed to track patient status over time, such that the items can be used repeatedly and provide an estimate of the patients’ “current” status. Thus, the items refer to a 30-day time period (i.e., “in the past 30 days,”). We only included behaviors that could change from time to time (i.e., historical items were excluded). Scores on the 17 items are summed to create a total score. A total score of nine or more indicates positive opioid misuse. In the current sample, the standardized alpha coefficient indicated good levels of reliability (α = 0.78).

### 2.4. Statistical Analyses

Descriptive statistics were calculated for the sample and study variables. Continuous variables are expressed as means and standard deviations and categorical variables are expressed as numbers and rates. The internal consistency of each instrument was assessed by calculating Cronbach’s standardized alpha coefficient for the sample. Cronbach’s alpha is used to estimate the proportion of variance that is systematic or consistent in a set of test items. We analyzed correlations between the observed variables included in the model. Finally, the hypothetical model was tested via Structural Equation Modelling (SEM) using LISREL 8.80 software (Scientific Software International Inc., 7383, Loncolnwood, IL, USA). A prior check of the data showed that some of the variables were not normally distributed. Thus, we used the Maximum Likelihood estimation method because it is effective for any data distribution if the analyses are performed on covariance matrices and the matrix of fourth-order moments is provided [57]. The following goodness-of-fit indexes were used: the Satorra–Bentler chi-square, the root mean-square error of approximation (RMSEA), the Comparative Fit Index (CFI), and the Non-normed fit index (NNFI). The Satorra–Bentler chi-square is a chi-square fit index that corrects the statistic under distributional violations. To reduce the sensitivity of chi-square to sample size, the index is divided by the degrees of freedom [58]. Ratios of 2 or less are indicative of an acceptable fit of the model [59]. The RMSEA is an absolute misfit index: The closer to zero, the better the fit. Values less than 0.08 indicate an adequate fit [60,61]. The CFI and the NNFI range between 0 and 1: The closer to 1, the better the fit [61]. Pain acceptance was the exogenous variable in the model. The endogenous variables were as follows: anxiety and depression symptoms, pain intensity, pain catastrophizing, and opioid misuse.

## 3. Results

### 3.1. Participants

The final sample comprised 140 chronic pain patients (115 women and 25 men) attending two hospitals. All participants were receiving pharmacological treatment, including opioid analgesics. Mean age was 59.24 years (SD = 9.77) and average pain duration was 17 years (SD= 13.4). The International Association for the Study of Pain classification was applied to determine the type of chronic pain [62]: chronic primary pain (56.4%), chronic secondary musculoskeletal pain (35.7%), chronic neuropathic pain (4.3%), chronic postsurgical or posttraumatic pain (2.9), and chronic secondary headache or orofacial pain (0.7%). At the time of the study, 66.4% of the participants were married, 41.4% were retired, 17.1% were unemployed, 22.9% were homemakers, 55% had completed primary education, and 29.3% had completed secondary education. Tramadol (35.7%), oxycodone (15%), and fentanyl (14.3%) were the most frequently used opioid analgesics.

### 3.2. Descriptive Statistics

Table 1 shows the mean scores, standard deviations, and correlation coefficients for all measures.

The guidelines proposed by Cohen [63] were used to assess correlations. As shown in Table 1, we found a high negative association between pain acceptance and depression symptoms, a medium negative correlation between pain acceptance and anxiety symptoms, catastrophizing, and pain intensity, and a low negative correlation between pain acceptance and opioid misuse. In contrast, we found a high positive significant correlation between opioid misuse and depression and anxiety symptoms, and a medium positive significant correlation between opioid misuse and pain catastrophizing. Surprisingly, the correlation between opioid misuse and pain intensity did not reach significance.

### 3.3. Structural Equation Modeling

Table 2 shows the standardized coefficients of the initial model.

In order to obtain a parsimonious model of the relationship between the variables and following the recommendations of the Lagrange Multiplier Test [59], we deleted all the non-statistically significant paths of the initial model. Thus, we excluded all paths from pain intensity and pain catastrophizing to prescription opioid misuse.

**Figure 1:**
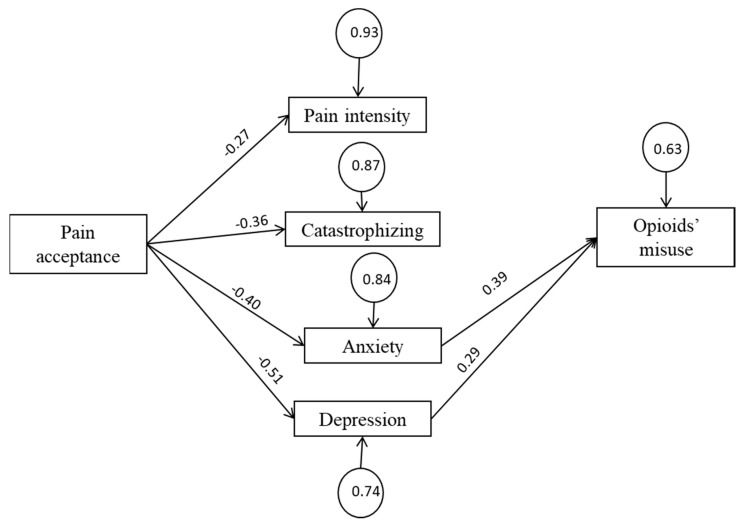
Final model.

Figure 1 represents the final model. All path coefficients were statistically significant (*p* < 0.05). The goodness-of-fit indexes calculated for the SEM indicate that the estimated model provides a good fit to the data (*χ*^2^(df) = 5.18 (6), *p* = 0.52; RMSEA = 0.00; NNFI = 1.00; CFI = 0.99). Figure 1 shows the standardized *Beta* (*β*) and *Gamma* (*γ*) coefficients, which can be interpreted as follows: *Beta* indicates that a change unit in an endogenous variable is associated with beta-change units in another endogenous variable, while all other variables remain constant. *Gamma* indicates that a change unit in an exogenous variable (pain acceptance) is associated with gamma-change units in an endogenous variable.

Rectangles represent observed variables, circles represent standardized error variances, arrowed lines represent presumed causal paths, values above the arrowed lines represent standardized *γ* and *β* coefficients (*p* < 0.05). Goodness-of-fit indexes of the tested models are as follows: *χ*^2^(df) = 5.18 (6), *p* = 0.52; RMSEA = 0.00; NNFI = 1.00; CFI = 0.99.

As expected, pain acceptance had four significant and negative path coefficients to pain intensity, pain catastrophizing, depression, and anxiety. However, only depression and anxiety symptoms yielded significant path coefficients to opioid misuse.

## 4. Discussion

Chronic pain is one of the most significant risk factors for opioid misuse [64]. The majority of people who misuse opioids were initially prescribed these drugs for chronic pain and, over time, departed from the initial medical directions [65,66]. Thus, it is relevant to investigate the role of the psychological factors involved in adjustment to chronic pain and in opioid misuse. There is ample evidence of an association between psychological flexibility processes, particularly pain acceptance, and better adjustment to pain [12,28,29] and preliminary evidence of an association between pain acceptance and a lower risk of opioid misuse in chronic pain patients [39,40,41]. Thus, the present study tested a hypothetical model in which pain acceptance would regulate the experience of pain, including pain intensity, catastrophic pain-related thoughts and emotions, and anxiety and depression. All of these factors are associated with opioid misuse. The results partially supported the postulated model. In particular, a significant association was found between higher pain acceptance and lower pain intensity, lower pain catastrophizing, and lower levels of anxiety and depression. Only higher levels of anxiety and depression were significantly associated with increased opioid misuse. These results are in line with a well-documented finding in the literature: Pain acceptance, defined as the response to pain-related experiences without attempts at control or avoidance and engaging in normal life activities even if pain is present [42], effectively regulates the experience of pain [12,28,29] and, indirectly, opioid misuse through anxiety and depression.

Interestingly, we found no association between self-reported pain intensity and opioid misuse. This finding is compatible with those of previous cross-sectional and longitudinal studies [67,68,69,70], which found that self-reported pain intensity contributed minimally to opioid craving. This variable is associated with prescription opioid misuse in chronic pain patients receiving long-term opioid therapy [67,71]. A recent longitudinal study found that the association between pain intensity and opioid misuse disappeared when controlling for negative affect and pain catastrophizing [72]. Instead, and in line with our results, a significant association has been found between the severity of prescription opioid misuse and self-reported anxiety and depression [67]. These results challenge the intuitive assumption that patients crave opioid medication due to pain and the relief that they expect to obtain, and instead suggest that opioid misuse is aimed at mitigating unpleasant emotional states.

Several studies have found an association between general measures of prescription opioid misuse and pain catastrophizing, defined as ruminating thoughts about the serious threat that pain represents and the lack of personal resources for coping with it [73,74,75,76,77]. Furthermore, an association has been found between increased pain catastrophizing and an increased likelihood of running out of opioid medication early, even after controlling for the patients’ levels of pain intensity and negative affect [72]. Pain catastrophizing has also been associated with increased cravings for prescription opioids [78]. This study found no evidence of an association between pain catastrophizing and opioid misuse. This finding is similar to that of a previous study [79]. The authors explained the discrepancy between the results and those of previous research in terms of the composition of their sample, which they judged to be more heterogeneous and representative of the population of patients treated at a tertiary pain clinic than samples investigated in previous studies. The discrepancy between our results and those reported in previous studies could be due to the assessment tool we used to measure pain catastrophizing. Previous studies have used the Pain Catastrophizing Scale [80], whereas we used the 2-item CSQ [50]. Although these items proved to be valid and reliable, as their authors recognize, they do not completely capture the entire content domain of the catastrophizing construct, which comprises the aforementioned dimensions of rumination, magnification, and helplessness [80].

The results of the present study suggest that anxiety and depression are associated with the misuse of prescription opioids in patients receiving opioid therapy. The relationship between anxiety and depression and substance abuse in general [81] and opioid misuse in particular is a well-documented finding [82,83]; there is also considerable evidence on the comorbidity of chronic pain and mood disorders [84]. Previous studies have consistently found that patients with high levels of anxiety and depression receiving opioid treatment for chronic pain are at increased risk of developing opioid misuse [80,81,82,83,84,85,86,87,88,89,90]. In fact, theories of addiction propose that escape from and avoidance of negative affect is the predominant motive for addictive drug use [91,92]. This perspective suggests that the mechanism sustaining opioid addiction could be a learned association between opioids and relief from an existing dysphoric state, which is formed and maintained by negative reinforcement [93,94]. In order to understand the mechanisms underlying the association between opioid-related and anxiety-related disorders, recent research has addressed transdiagnostic vulnerability factors [95], and specifically, anxiety sensitivity [96], emotion dysregulation [97], pain-anxiety [89], distress intolerance [98], and acceptance [39,40,41] as in the present study. All these factors refer to maladaptive responses to emotional states that are mainly characterized by avoidance, although there is some degree of overlap between them. Thus, there is a need for these results to be integrated into an overarching theoretical model. In this regard, the Psychological Flexibility Model [14] is promising. Until now, most related studies have focused on acceptance and experiential avoidance. Future research could address the role of all the elements of the model in relation to co-morbid anxiety and depression and opioid misuse in chronic pain patients.

A research study [99] found that some of the factors associated with opioid misuse are similar to those associated with opioid prescription. In a sample of patients with noncancer chronic pain, the participants who were prescribed opioids were older, reported higher levels of pain intensity and depressive symptoms and reported lower levels of pain-acceptance than those who had not been prescribed opioids. It could be the case that physicians prescribe opioids more frequently to those patients who are at greater risk of opioid misuse. A future line of research would be to simultaneously study the factors involved in the prescription of opioids and the factors involved in their misuse in the same group of participants.

The current study has limitations. Firstly, the exclusive reliance on self-report measures could have influenced the results due to shared methodological variance. Secondly, the cross-sectional nature of the design precludes drawing causal inferences. Thirdly, the capacity to generalize the results of this study might be limited by the characteristics of the sample: Women were overrepresented; all the participants had long-lasting musculoskeletal pain; their level of education level was low, and most of them were no longer active workers. Finally, future studies could investigate the differential effect of different types of opioid medications.

This study has relevant clinical implications. Firstly, in order to halt the alarming spread of opioid misuse, anxiety, depression, and pain acceptance should be assessed in patients as a priority when prescribing opioids. In addition, patients with significant levels of depression and anxiety and low levels of pain acceptance should be carefully monitored by clinicians. Secondly, since our findings and those of other studies suggest that anxiety and depression are related to opioid misuse, Cognitive Behavioral Therapy [11] and, specifically, ACT [100], which promotes pain acceptance, appear to be particularly appropriate as adjunctive therapy to standard treatment for these patients.

## 5. Conclusions

Patients who are able to accept their pain-related sensations, thoughts, and emotions will experience lower levels of anxiety and depression, and thus will be at a lower risk of opioid misuse. Anxiety, depression, and pain acceptance should therefore be routinely assessed before opioids are prescribed. Psychological interventions may be needed when depression, anxiety, and low levels of pain acceptance are detected. Clinicians should be extremely cautious when prescribing opioids to these patients.

## Figures and Tables

**Table 1:** Descriptive statistics and correlations between measures.

	*M*	SD	Range	1	2	3	4	5	6
1. Pain acceptance	17.39	13.78	0–46	1					
2. Anxiety	19.80	5.77	7–28	−0.37 **	1				
3. Depression	15.77	5.08	7–27	−0.55 **	0.55 **	1			
4. Catastrophizing	5.47	2.18	2–8	−0.48 **	0.46 **	0.62 **	1		
5. Pain intensity	6.94	1.67	0–10	−0.35 **	0.20 *	0.27 **	0.29 **	1	
6. Opioid misuse	15.55	8.64	0–39	−0.28**	0.57 **	0.52 **	0.44 **	0.16	1

*Note*: *M* = Means; SD = Standard Deviations; Range = Minimum and Maximum scores. ** *p* < 0.001: * *p* < 0.05 (Pearson’s correlations).

**Table 2:** Initial model. Standardized gamma and beta coefficients.

	Pain Acceptance	Opioid Misuse
	*γ*	*β*
Pain intensity	−0.27 *	0.06
Pain catastrophizing	−0.36 *	0.12
Anxiety	−0.40 *	0.36 *
Depression	−0.51 *	0.25 *

* *p* < 0.05.

## Data Availability

Not applicable.
